# Insights into the Behavior of Triple-Negative MDA-MB-231 Breast Carcinoma Cells Following the Treatment with 17β-Ethinylestradiol and Levonorgestrel

**DOI:** 10.3390/molecules26092776

**Published:** 2021-05-08

**Authors:** Sebastian Simu, Iasmina Marcovici, Amadeus Dobrescu, Daniel Malita, Cristina Adriana Dehelean, Dorina Coricovac, Flavius Olaru, George Andrei Draghici, Dan Navolan

**Affiliations:** 1 Research Center for Pharmaco-Toxicological Evaluations, Faculty of Pharmacy, “Victor Babes” University of Medicine and Pharmacy, Eftimie Murgu Square No. 2, RO-300041 Timisoara, Romania; simu.sebastian@umft.ro (S.S.); iasmina.marcovici@umft.ro (I.M.); cadehelean@umft.ro (C.A.D.); dorinacoricovac@umft.ro (D.C.); draghici.george-andrei@umft.ro (G.A.D.); 2 Faculty of Pharmacy, “Victor Babes” University of Medicine and Pharmacy, Eftimie Murgu Square No. 2, RO-300041 Timisoara, Romania; 3 Faculty of Medicine, 2nd Department of Surgery, “Victor Babes” University of Medicine and Pharmacy, Eftimie Murgu Square No. 2, RO-300041 Timisoara, Romania; 4 Faculty of Medicine, Department of Radiology, “Victor Babes” University of Medicine and Pharmacy, Eftimie Murgu Square No. 2, RO-300041 Timisoara, Romania; 5 Faculty of Medicine, Department of Obstetrics and Gynecology, “Victor Babes” University of Medicine and Pharmacy, Eftimie Murgu Square No. 2, RO-300041 Timisoara, Romania; olaru.flavius@umft.ro (F.O.); navolan@umft.ro (D.N.)

**Keywords:** triple-negative breast cancer, 17β-Ethinylestradiol, Levonorgestrel, MDA-MB-231 cells, proliferation, migration

## Abstract

Oral contraceptives (OCs) are widely used due to their efficiency in preventing unplanned pregnancies and treating several human illnesses. Despite their medical value, the toxicity of OCs remains a public concern. Previous studies indicate the carcinogenic potential of synthetic sex hormones and their link to the development and progression of hormone-dependent malignancies such as breast cancer. However, little is known about their influence on the evolution of triple-negative breast carcinoma (TNBC), a malignancy defined by the absence of estrogen, progesterone, and HER2 receptors. This study reveals that the active ingredients of modern OCs, 17β-Ethinylestradiol, Levonorgestrel, and their combination induce differential effects in MDA-MB-231 TNBC cells. The most relevant behavioral changes occurred after the 24 h treatment with 17β-Ethinylestradiol, summarized as follows: (i) decreased cell viability (64.32% at 10 µM); (ii) cell roundness and loss of confluence; (iii) apoptotic aspect of cell nuclei (fragmentation, membrane blebbing); and (iv) inhibited cell migration, suggesting a potential anticancer effect. Conversely, Levonorgestrel was generally associated with a proliferative activity. The association of the two OCs exerted similar effects as 17β-Ethinylestradiol but was less effective. Further studies are necessary to elucidate the hormones’ cytotoxic mechanism of action on TNBC cells.

## 1. Introduction

Oral contraception is one of the most popular and efficient birth control methods, being used by a significant number of women (hundreds of millions) of reproductive age worldwide [1,2]. From their discovery to the current days, contraceptive pills continuously evolved regarding their active ingredients, dosage, and therapy regimens [2,3]. Although in early formulations estrogen was the only active constituent [2], in modern pills it is associated with progestin in various combinations and doses [3]. 17β-Ethinylestradiol (EE), which was synthesized in the 1930s by the substitution of estradiol at C17 with an ethinyl group [4], is the main estrogen found in the past and current oral contraceptives (OCs) [2,3]. Levonorgestrel (LNG) is a second-generation synthetic progestogen and the biologically active isomer of the racemic mixture of norgestrel [5,6], possessing two current indications: (i) as the first-line oral emergency contraceptive and (ii) as a long-term drug for birth control in combination with estrogen [5].

Besides contraception, OCs are addressed to the treatment of other health issues (e.g., dysmenorrhoea, irregular menstruation, fibroids, endometriosis, rheumatoid arthritis, multiple sclerosis, migraine) [7,8]. Despite their therapeutic value, the use of OCs is associated with minor (e.g., nausea, headaches, abdominal cramping, breast tenderness, weight gain, acne, mood changes, bleeding irregularities) and major adverse reactions (e.g., thromboembolic events, myocardial infarction, stroke) [7,9]. Additionally, several concerns were expressed regarding the OCs’ carcinogenic potential [1]. A controversy in this area of research is the tissue-type dependent activity of OCs which decreases the risk of endometrial, ovarian, and colorectal cancers while increasing the risk of breast, cervical, and liver cancers [10,11].

Breast cancer (BC) is ranked in the top three of the most common malignancies worldwide, as well as the most recurrent type of cancer in women. Statistically, one in eight to ten women develops BC during their lifetime [12]. Breast cancer is a heterogeneous [13] and multifactorial disease with various factors contributing to its occurrence, including age, family history, demography, lifestyle, and exposure to sex hormones [14,15]. BC is acknowledged to be a hormone-dependent disease. The implication of endogenous and exogenous sex hormones to the development of cancer stands in their ability to maintain high mitotic rates and increased cell division [16]. Up to date, experimental and clinical data have identified the crucial role that estrogen plays in the proliferation and progression of BC [17]. Although the precise carcinogenic mechanisms of estrogen are not completely understood [18], it is well known that it regulates human health and diseases by binding to the specific estrogen receptors alpha (ER-α) and beta (ER-β) [19]. In the case of breast cancer, estrogen primarily acts through ER-α [18]. The clinical significance of ER-β remains unelucidated [13], but several data indicate its tumor-suppressive properties [20,21]. The ER signaling pathway promotes unequal rates of division and apoptosis in cancer cells, supporting the pro-survival signals over the pro-death ones [17]. Although the major focus has been on estrogens, progesterone and progestin exposure are increasingly recognized as instrumental in the evolution of BC [22]. In a concise review, Hilton and colleagues summarized the following facts: (i) women exposed to progestin presented an elevated risk to develop BC as compared to women taking estrogen-only formulations, and (ii) the tumors developed following the administration of synthetic progestins tended to be more aggressive [17]. Progestins are able to stimulate the proliferation and to inhibit apoptosis in BC cells, as well as to reverse the antitumor effect of the breast cancer hormonal therapy [17]. However, the activity of progesterone and synthetic progestins on breast cancer cells is rather controversial. Not only can they stimulate the proliferation of breast cancer cells, but also inhibit it depending on the cellular context and cancer stage [23]. Similarly with estrogen, progesterone binds to its nuclear receptors which are expressed in two different isoforms—progesterone receptor (PR) A and B. The PRs are similarly expressed in the normal breast epithelium, while there is an imbalance in their levels which occurs during the early breast cancer development [17]. The interaction between estrogen and progesterone and their receptors has emerged as a key player in the BC development and progression [16].

BC covers a group of many biologically distinct subtypes presenting differences as respects their response to various treatment modalities, as well as clinical outcome [24]. The expression of estrogen receptors (ERs), progesterone receptors (PRs), and the human epidermal growth factor receptor 2 (HER2) is commonly used for the classification of invasive BC [14]. Breast cancer is divided into the following clinically relevant molecular subtypes: luminal A and B (ER/PR+ or both, HER2-); non-luminal (ER-, PR-, HER2+); basal-like or triple-negative (ER-, PR-, HER2-) [12]. From the total of breast carcinomas, 70 to 75% are hormone receptor-positive cancers [20,25], the presence of ERs and/or PRs being recognized as a favorable prognostic biomarker [17]. HER2 occurs in 15–20% of BC patients, being a predictive biomarker for poor disease prognosis since its overexpression leads to uncontrolled cell proliferation. However, its presence indicates a positive response to HER2-targeted treatment [26]. The triple-negative breast cancer (TNBC) is characterized by an aggressive behavior, with early relapse and metastatic spread to several organs such as lung, liver, and brain [27], being responsible for more than 50% of the breast cancer-related deaths [21]. TNBC is uniquely defined by the absence of hormone receptors (ER-α/PRs) and negativity for HER2, accounting for approximately 10–15% of all breast cancer diagnoses [27,28]. However, several markers such as ER-β, G-protein coupled estrogen receptor 1 (GPER-1), and the estrogen-related receptors (ERRs) are frequently expressed, which might be responsible for the estrogen-sensitivity of TNBC [21]. Other features of TNBC include the high rate of chromosomal mutations and mitotic count, mutations of tumoral protein p53 and of human tumor suppressor gene BRCA1, as well as its resistance to therapy [29]. Clinical aspects of TNBC include high invasive potential, susceptibility to recurrence, the poorest prognosis among all BC subtypes and limited treatment options [27,30,31] as the lack of ERs, PRs, and HER2 expression leads to insensitivity of TNBC tumors to hormonal or HER2-targeted therapies [30]. Recent papers indicate a link between OCs and TNBC development, but this subject is far from being elucidated [29].

This paper aims to offer several insights regarding the role of two oral contraceptives—17β-Ethinylestradiol and Levonorgestrel in triple-negative breast cancer by assessing in vitro their impact on the proliferation and the metastatic behavior of the highly aggressive and invasive triple-negative breast cancer cell line MDA-MB-231.

## 2. Results

### 2.1. Cell Viability Evaluation

**Figure 1:**
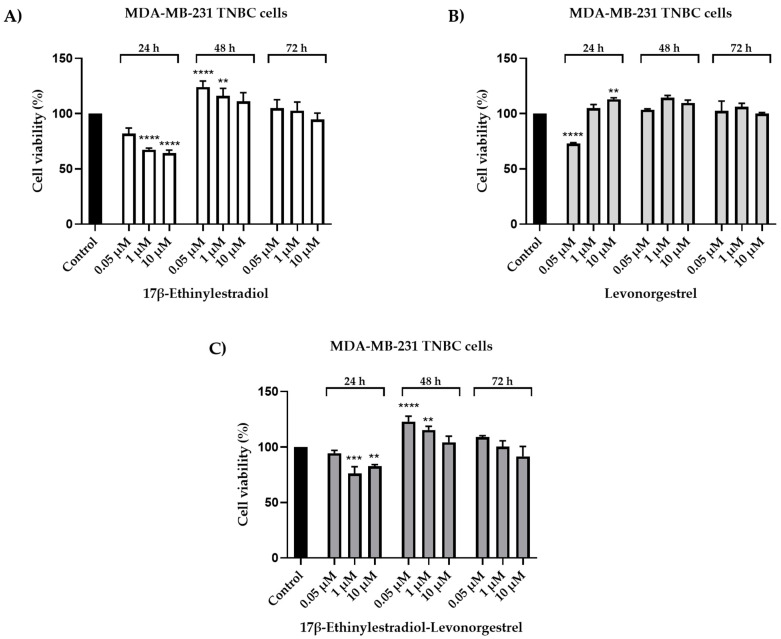
In vitro assessment of: (**A**) 17β-Ethinylestradiol, (**B**) Levonorgestrel, and (**C**) 17β-Ethinylestradiol-Levonorgestrel (0.05, 1, and 10 µM) impact on the viability of MDA-MB-231 triple-negative breast cancer cells after 24, 48, and 72 h of treatment by applying the MTT assay. The data are presented as cell viability percentage (%) normalized to control (untreated) cells and are expressed as mean values ± SD of three independent experiments performed in triplicate. To identify the statistical differences between the control and the treated group, the one-way ANOVA analysis was conducted, followed by the Dunnett’s multiple comparisons post-test (** *p* < 0.01, *** *p* < 0.001, and **** *p* < 0.0001).

Since the impact of the prolonged use of oral contraceptives on the evolution of triple-negative breast cancer is debatable, in the present study, the MDA-MB-231 breast cancer cell line was exogenously treated with 17β-Ethinylestradiol (EE), Levonorgestrel (LNG), and 17β-Ethinylestradiol-Levonorgestrel (EE-LNG) solutions in DMSO. Increasing concentrations (0.05, 1, and 10 µM) were tested for three-time intervals: 24, 48, and 72 h. The induced cytotoxic effect was time-dependent. Our results suggest a significant antiproliferative activity in the case of EE after the 24 h treatment, which induced a dose-dependent reduction of MDA-MB-231 cells’ viability, the lowest percentage of viable cells (64.32%) being calculated for the highest concentration tested—10 µM. As regards the treatment for 48 and 72 h with EE, the results were very different as compared to the ones calculated for 24 h, being noticed a stimulatory effect of EE (the lowest dose—0.05 induced the highest increase in cells viability percentage) (Figure 1A).

The 24 h treatment with LNG significantly reduced the cell viability at low concentrations (0.05 µM–72.79%), while increasing it at high concentrations (10 µM–113%). After longer treatments (48 and 72 h), LNG stimulated the proliferation of MDA-MB-231 cells (all cell viability percentages were over 100%) (Figure 1B). A considerable inhibition of cell proliferation has been observed after the 24 h treatment with EE-LNG (1 µM–76.08%; 10 µM–82.67%). Although the 24 h results indicate a cytotoxic effect, a stimulatory effect has been noticed after the 48 h treatment. The effect was dose-dependent. The cell viability percentages assessed after the 72 h treatment with EE-LNG were similar to control. However, a slight decrease was induced by EE-LNG 10 µM (91.59%) (Figure 1C).

### 2.2. Cellular and Nuclear Morphology Assessment

**Figure 2:**
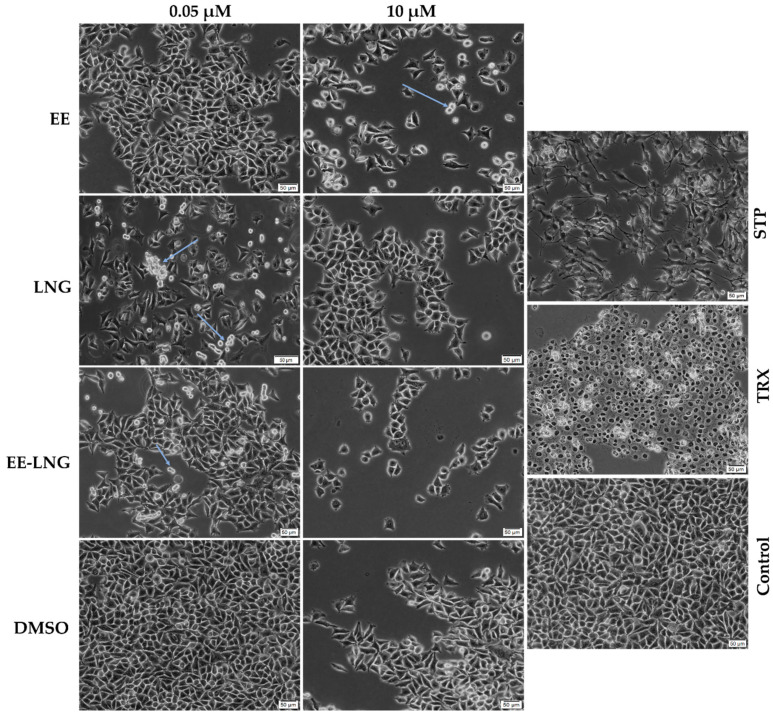
Morphological aspect of the MDA-MB-231 cells after the treatment with EE, LNG, EE-LNG, and DMSO (0.05 and 10 µM) solutions for 24 h. The arrows indicate the round and detached cells noticed following the treatment with OCs. The morphological changes induced by Staurosporine (STP) 5 μM selected as an indicator for apoptosis and Triton X-100 (TRX) 0.5% used as an indicator for necrosis are presented as well. The pictures were taken 24 h post-treatment. The scale bars represent 50 µm.

As the most relevant results induced by oral contraceptives on the cellular viability were recorded after 24 h of treatment, photographs of the MDA-MB-231 cells and nuclei were taken to assess the cytotoxic impact of EE, LNG, and EE-LNG solutions in terms of morphological changes. The lowest (0.05 µM) and the highest (10 µM) concentrations were used for this experiment. The pro-apoptotic agent Staurosporine (STP) 5 μM and the pro-necrotic agent Triton-X 100 (TRX) 0.5% were selected as positive controls for cytotoxicity. The pictures suggest a loss of cell confluence after the treatment with EE, LNG, and EE-LNG solutions, as compared to the control and DMSO. The roundness and non-adherence of the cells (indicated by arrows) noticed after the stimulation with OCs suggest cell death and are more prominent in the case of EE 10 µM, LNG 0.05 µM, and EE-LNG 0.05 µM. However, contrary to STP and TRX, the contraceptives induced no visible changes in the morphological aspect of the viable adherent cells (Figure 2).

**Figure 3:**
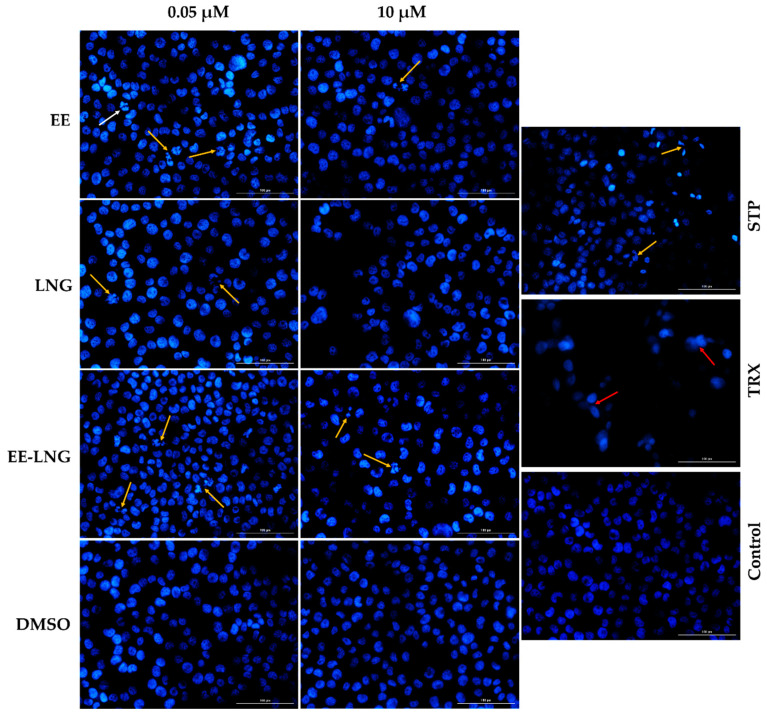
Cell nuclei staining using Hoechst 33342 in MDA-MB-231 cells after the treatment with EE, LNG, EE-LNG, and DMSO (0.05 and 10 µM) solutions for 24 h. The pictures were taken 24 h post-treatment. Staurosporine (STP) solution 5 μM was used as a positive control for apoptotic changes at the nuclear level and Triton X-100 (TRX) solution 0.5% was used as an indicator for necrosis. The yellow arrows indicate the apoptotic cells with nuclear fragmentation, the red arrows indicate necrotic nuclei, and the white arrow shows the nucleus of a cell undergoing mitosis. The scale bars represent 100 µm.

In addition to the viability and cell morphology investigations, the Hoechst 33342 nuclear staining was performed to evaluate the potential cellular death mechanism induced by EE, LNG, and EE-LNG after 24 h of treatment. The interpretation of the results was performed according to Crowley et al. [32]. Regarding the aspect of the cell nuclei, relevant changes were noticed after the stimulation with EE, LNG, and EE-LNG at 0.05 µM and 10 µM such as nuclear fragmentation, membrane blebbing, and apoptotic bodies indicated by yellow arrows (Figure 3). The condensation of chromatin is suggested by the intense blue color of the nuclei. No signs of necrosis were noticed after the cells’ treatment with oral contraceptives. The solvent induced no changes in the nuclei morphology that could suggest cell death. The white arrow (EE 0.05 µM) indicates the nucleus of a cell undergoing mitosis (Figure 3).

### 2.3. Wound Healing Assay and Quantitative Polymerase Chain Reaction (RT-qPCR)

**Figure 4:**
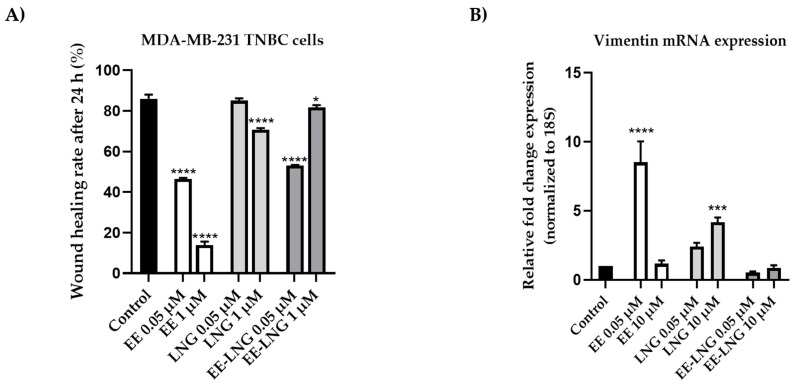
(**A**) The impact of EE, LNG, and EE-LNG 0.05 and 1 µM on the migratory capacity of the MDA-MB-231 breast cancer cell line by applying the wound healing assay. The bar graphs are expressed as percentage of wound closure after 24 h compared to the initial surface. (**B**) The influence of EE, LNG, and EE-LNG (0.05 and 1 µM) on the Vimentin mRNA expression was recorded after a 48 h treatment. The data are presented as relative fold change expression normalized to 18S (used as housekeeping gene). All results are expressed as mean values ± SD of three independent experiments performed in triplicate. The statistical differences between the control and the treated group were identified by applying the one-way ANOVA analysis, followed by the Dunnett’s multiple comparisons post-test (* *p* < 0.05, *** *p* < 0.001, and **** *p* < 0.0001).

To determine the impact of contraceptives on the migratory ability of MDA-MB-231 breast cancer cells, the scratch assay was applied. For this experiment, two concentrations (0.05 µM and 1 µM) were selected for every sample. Untreated cells (control) presented the highest migration rate (85.97%), followed by the cells treated with LNG 0.05 µM (85.01%) and EE-LNG 1 µM (81.67%). The most potent anti-migratory effect was noticed in the case of EE 0.05 µM and 1 µM with wound healing rates of 46.38% and 13.91%, respectively. Significant inhibition of the wound healing rate was observed in the cells treated with LNG 1 µM (70.73%) and EE-LNG 0.05 µM (53.04%), respectively. The data are presented in Figure 4A.

As a supplement to the results obtained from the wound healing assay regarding the impact of OCs on the invasiveness of MDA-MB-231 TNBC cells, the changes in the expression of vimentin were analyzed via the RT-qPCR technique after a 48 h stimulation period with EE, LNG, and EE-LNG solution. The lowest and the highest concentrations tested (0.05 and 10 µM) were selected for this experiment. When compared to control (untreated cells), OCs induced relevant changes in the expression of vimentin as follows: (1) EE and LNG up-regulated vimentin mRNA expression, significant changes being noticed for EE 0.05 µM (ANOVA, *p* < 0.0001) and LNG 10 µM (ANOVA, *p* < 0.001); and (2) although the differences between control and EE-LNG groups were not statistically significant, the association of EE with LNG decreased the gene expression at both concentrations (Figure 4B).

## 3. Discussion

The present study was conducted to investigate the impact of the two synthetic sex hormones 17β-Ethynilestradiol (EE) and Levonorgestrel (LNG) and their combination (EE-LNG) on the MDA-MB-231 triple-negative breast carcinoma cells’ behavior in order to gather insights regarding their role in breast cancer development, a subject unelucidated at present. The major findings in this direction indicated a different behavior of MDA-MB-231 cells dependent on test compound, concentration and treatment duration, as follows: (i) EE treatment determined a dose- and time-dependent cytotoxicity at the earliest time point—24 h characterized by reduced cell viability percentage (Figure 1A) associated with cellular morphological changes (Figure 2) and apoptotic features (Figure 3), whereas longer treatments (48 and 72 h) induced a proliferative effect (Figure 1A); (ii) LNG treatment proved to be cytotoxic only at the lowest concentration tested—0.05 µM after 24 h (with morphological changes and apoptotic specific signs) (Figure 1B or Figure 2 and Figure 3), higher concentrations and longer treatments being associated with a stimulatory effect; and (iii) EE-LNG treatment presented a trend similar to the one described for EE, a significant decrease of cell viability percentage being recorded only after 24 h treatment, with the mention that EE alone proved to be more active than EE-LNG (Figure 1C or Figure 2 and Figure 3). In addition, the test compounds (EE, LNG, and EE-LNG—0.05 and 1 µM) exerted an inhibitory effect on MDA-MB-231 cells migratory capacity, the most notable inhibitory effect being attributed to EE treatment, and interfered with mesenchymal (vimentin) marker mRNA expression, as follows: EE-LNG—down-regulated vimentin expression, whereas in the case of EE and LNG, the mRNA expression of vimentin was up-regulated.

Sex hormones play a substantial role in the etiopathogenesis and progression of various cancers, including breast cancer [33]. A strong link has been established between the endogenous levels of sex steroids and breast cancer risk [33,34]. Furthermore, oral contraception, which consists of synthetic estrogens and progestins [3], has been widely suspected to co-participate in the development and progression of BC [35]. Mechanistically, the female sex hormones, estrogen and progesterone, promote the growth and development of breast tumors through their specific nuclear receptors, increasing the number and mitotic rate of the cells [33]. Estrogens act by binding to the estrogen receptors ER-α and ER-β which play contradictory roles in the proliferation of BC cells [36]. ER-α elicits a stimulatory activity on the progression and metastasis of breast cancer [36], while ER-β, on the other hand, has tumor-suppressive properties [20,21]. Progesterone acts via two receptor isoforms PR-A and PR-B [17]. A vast majority of breast cancers are hormone receptor-positive [20,25], ERs and PRs being pivotal biomarkers for cancer prognostic and response to therapy [17]. Triple-negative breast cancer is a uniquely featured malignancy, lacking the expression of ER-α, PRs, and HER2 [28,31]. However, the TNBC cells frequently express ER-β, G-protein coupled estrogen receptor 1 (GPER-1), estrogen-related receptors (ERRs) [21], and membrane PR-α (mPR-α) receptors which are unrelated to the nuclear PRs [37,38]. Furthermore, it is possible that some other unidentified receptors bind estrogen and progesterone in TNBC, which can be easily identified with a computational approach called inverse docking that successfully enables the identification of protein targets involved in molecular mechanisms of side effects and anticarcinogenic action of compounds [39,40].

Regarding TNBC, estrogen can trigger tumor-promoting effects or act as an antitumoral agent through ER-β activation [21]. Although the function of progesterone in TNBC has not been completely identified yet, evidence indicates that it suppresses the growth and invasion of TNBC cells expressing mPR-α receptors [37,38]. Most of the studies conducted to evaluate the implication of sex hormones in the evolution of triple-negative breast cancer employed endogenous hormones (17β-estradiol [41,42], and progesterone [38]) as test compounds, while the data regarding the effects of synthetic hormones used as oral contraceptives remain poor.

The hypothesis that led to the implementation of the present study was that some active ingredients present in contraceptive pills might impact the behavior of human triple-negative breast cancer cells. Accordingly, the MDA-MB-231 cell line was chosen as an in vitro model for triple-negative breast carcinoma, while EE and LNG—the most widely employed contraceptive agents nowadays [2,43], were selected as representatives for modern OCs. MDA-MB-231, which is derived from a pleural effusion from a patient diagnosed with BC [44], is one of the most commonly studied cell lines [44] and a good representative for TNBC since the cells lack ER, PR, and HER2 biomarkers [45]. Furthermore, the cells are characterized by low Ki67, E-cadherin, claudin-3, -4 and -7 expression [46], as well as mutated tumor protein p53 [45]. Their response to chemotherapy is intermediate [46], MDA-MB-231 being a highly aggressive and invasive breast cancer model, both in vitro and in vivo [45]. The concentrations tested in this study (0.05, 1, and 10 µM) were selected based on a revision of the literature regarding the doses of EE, LNG, and EE-LNG tested previously in vitro [10,47].

Regarding cell proliferation, the treatment with EE, LNG, and EE-LNG led to time- and test compound-dependent results. After 24 h of stimulation, the MDA-MB-231 cells manifested a significant sensitivity to OCs and their combination. A decreasing trend has been noticed in the case of EE, the most potent antiproliferative effect being induced at the highest concentration of 10 µM when the cell viability was reduced to 64.32%. However, an opposite effect was induced by LNG, the cell viability increasing in a dose-dependent manner from 72.79% at 0.05 µM to 113% at 10 µM. In the case of EE-LNG, a relevant reduction in cell viability was observed at 0.1 µM (84.32%), 1 µM (76.08%), and 10 µM (82.67%). The morphology of the cells was visibly altered when exposed to birth control ingredients individually or in combination for 24 h as compared to control cells, the cells becoming non-adherent and round (Figure 2).

The efficacy of high doses of synthetic estrogens in the treatment of advanced breast cancer has been already revised in the literature [48]. Estrogens act by binding to their receptors [36]. While ER-α is absent, the ER-β was detected in many TNBCs [49], including MDA-MB-231 breast cancer cells in low amounts [50]. Since ER-β is associated with decreased cancer proliferation and progression [49,51] and EE has an affinity for this receptor subtype [36], it can be assumed that the antiproliferative effect of EE that we noticed after the 24 h treatment might be the consequence of the EE-ER-β interaction. Similarly, the suppression of the TNBC growth induced by LNG at low concentrations can be associated with its action on the progesterone receptor α (mPRα) which is expressed on the membrane of MDA-MB-231 cells [38]. The suppressive activity of sex hormones on the proliferation of breast cancer cells has been previously mentioned. In a recent study performed by Susana and colleagues, the influence of EE and LNG at high and low concentrations on the proliferation of a breast cancer cell line (Hs 578 Bst) was investigated. The proliferation of breast cells decreased with the addition of Ethinylestradiol and Levonorgestrel at high doses (20, 40 and 80 µg/mL for EE; 0.05 mg/mL, 0.025 mg/mL and 0.0125 mg/mL for LNG). At blood level concentrations (600 pg/mL for EE and 3.04 ng/mL for LNG), a decrease in the cells’ proliferation followed the addition of LNG, as well as EE and LNG combined. However, the proliferation was increased by EE when added individually [52]. Likewise, Zhou et al. obtained a dose-dependent inhibition of the MDA-MB-231 cellular proliferation after their treatment with progesterone 20 ng/mL, 40 ng/mL, and 80 ng/mL [38].

In contrast to the observations from the 24 h treatment, after 48 h, a stimulatory effect was caused by all samples, while after 72 h the results were mostly similar to control. Estrogen is implicated in breast carcinogenesis via ER-dependent and -independent mechanisms [53]. The ER-independent pathway implies (i) the conversion of estrogens into catechols and quinones catalyzed by cytochrome P450 enzymes (e.g., CYP3A4) present in the mammary tissue [53] and (ii) the generation of reactive oxygen species (ROS) [54], both promoting the breast carcinogenicity [54]. A plausible explanation for our results could be the in vitro metabolization of EE to derivatives able to stimulate the mitotic rate of the MDA-MB-231 cells. Additionally, the modulation of proliferative and cell death genes by EE might be the cause of increased cell proliferation. A study by Merki-Feld and colleagues reveals an increased cell proliferation rate in two estrogen and progesterone receptor-positive BC cell lines (ZR75-1 and HCC1500) following a long-term regimen with Estradiol and Ethynilestradiol, as well as a negative influence of estrogens on the expression of proliferative and apoptotic biomarkers such as elevated anti-apoptotic protein Bcl-2, and proliferating cell nuclear antigen (PCNA) [55]. Similar to estrogens, progesterone and Levonorgestrel can be locally converted by the enzyme 5α-reductase to 5α-pregnanes (e.g., 5α-dihydroprogesterone and 5α-dihydrolevonorgestrel) which, at high concentrations, are able to stimulate mitosis and suppress apoptosis. 5α-reductase is highly expressed in both hormone-responsive and -unresponsive BC cell lines [56,57], including MDA-MB-231 [58] which explains the increased cell viability caused by LNG. Hasan et al. observed a dose-dependent stimulatory effect of estrogen and progesterone (1 pM, 1 nM, 100 nM, 1 µM, 10 µM) in MCF-7 cells after 24 h, while no concentration-dependent effects on the proliferation of MDA-MB-231 cells [47].

The decrease in cell viability after 24 h of treatment led to the implementation of the Hoechst 33342 staining assay to evaluate the potential death mechanism induced by OCs. The fragmentation of the nuclei and chromatin condensation induced by EE, LNG, and EE-LNG represent features of apoptotic cell death (Figure 3). Previous in vitro studies support the pro-apoptotic activity of estrogens [59]. In a study conducted by Lewis et al., the 72 h treatment with estradiol 1 nM increased the expression of pro-apoptotic proteins (Bax, Bak, Bim, Noxa, Puma, and p53), enhanced the release of cytochrome c and the cleavage of PARP in an ER–positive and estrogen-deprived breast cancer cell line (MCF-7:5C). The DAPI staining of the cell nuclei indicated apoptotic characteristics such as condensed chromatin, and nuclear fragmentation [60]. Recently, progesterone (20 µM) was shown to trigger apoptosis via the activation of caspase-8 in ovarian and endometrial cancer cells [61]. A similar effect was noticed for LNG. In an in vitro study, the apoptosis rate in endometrial cancer cells increased after the 24 h treatment with 5 × 10^−5^ M LNG [62]. A recent in vivo study showed that the oral administration of EE-LNG can modulate apoptosis marker genes, down-regulating the Bcl-2, and up-regulating the Caspase-1 and -3 expressions in female rats [63].

Taking into consideration the high metastatic potential of the MDA-MB-231 cells, another aspect that was investigated in this study is the impact of EE, LNG, and EE-LNG on their migratory capacity. Thus, a wound healing assay was performed. Since at the highest concentration of 10 µM, signs of cytotoxicity and apoptosis were observed, the concentrations selected for this assay were 0.05 and 1 µM. EE significantly inhibited the migration of the TNBC cells at both concentrations, with wound healing rates of 46.38% and 13.91%, respectively. LNG interfered with the cell migration only at the highest concentration tested, while EE-LNG considerably reduced the cell migration at 0.05 µM. No stimulatory effect was noticed after the 24 h treatment. Previous papers offer evidence that ER-β may act as an important factor that influences ER-negative breast cancer cell motility and invasiveness in response to estrogen [64]. For instance, Hinsche and colleagues demonstrated that the treatment with the ER-β selective estrogen agonists liquiritigenin and ERB-041 inhibited the migration of TNBC cell lines HCC1806 and HCC1937 co-cultured with MG63 osteoblast-like cells [49].

In the light of the results obtained when it was evaluated the impact of the test compounds on the MDA-MD-231 cells’ migratory capacity, it was also assessed the effect of EE, LNG, and EE-LNG on the vimentin mRNA expression, a mesenchymal marker that is associated with tumor invasion, metastasis and a poor prognosis in different types of cancers, including breast cancer. Vimentin contributes to the epithelial-to-mesenchymal transition (EMT) and cancer cell mechanics by mediating the cytoskeletal organization and focal adhesion maturation [65]. Our data indicate compound-dependent results. Individually, EE and LNG manifested a stimulatory effect after 48 h of treatment, while their association down-regulated vimentin mRNA expression (Figure 4B). Since in MDA-MB-231 breast cancer cells, the vimentin depletion has been associated with reduced proliferation and affected wound healing [65], it can be said that the 48 h treatment with EE-LNG positively impacted the invasiveness of the cells. Hernández-Vega et al. observed an elevation of the EMT biomarker vimentin in human glioblastoma cells induced by 17β-estradiol 10 nM after 24, 48, and 72 h treatments. Chun-Ling Lin and colleagues reported an increased expression of vimentin in progesterone-treated PR-transfected MDA-MB- 231 cells, data that support our findings [66].

## 4. Materials and Methods

### 4.1. Reagents

17β-Ethinylestradiol (≥98% purity), Levonorgestrel, phosphate saline buffer (PBS), trypsin-EDTA solution, dimethyl sulfoxide (DMSO), fetal bovine serum (FBS), penicillin/streptomycin mixture, and MTT reagent were purchased from Sigma Aldrich, Merck KgaA (Darmstadt, Germany). The cell culture medium, Dulbecco’s Modified Eagle Medium (DMEM–ATCC^®^ 30-2002™), was bought from ATCC (American Type Cell Collection, Lomianki, Poland). All reagents used in the present study were of analytical grade purity and for cell culture use.

### 4.2. Cell Line and Cell Growth Conditions

This study was performed using the MDA-MB-231 (ATCC HTB-26™) breast cancer cell line which was acquired from ATCC as a frozen vial. The cells were cultured in their specific growth medium—DMEM supplemented with 10% FCS and 1% antibiotic mixture (100 U/mL penicillin/ 100 µg/mL streptomycin). During the experiments, the cells were incubated in a humidified atmosphere (37 °C; 5% CO_2_).

### 4.3. Cell Viability Evaluation

The cell viability assessment was performed by the means of the MTT assay. Briefly, the cells were seeded in 96-well plates at a density of 10^4^ cells/200 µL/well and were treated with increasing concentrations (0.05, 1, and 10 µM) of Ethynilestradiol (EE), Levonorgestrel (LNG), and Ethynilestradiol-Levonorgestrel (EE-LNG) solutions for 24, 48, and 72 h. The solvent used to prepare the stock solutions was dimethyl sulfoxide (DMSO). After the stimulation period (24, 48, and 72 h), an amount of 100 µL of fresh media and 10 µL of MTT reagent was added in each well and the cells were incubated for 3 h at 37 °C. Finally, 100 µL of solubilization solution were added, the plate was kept at room temperature for 30 min, protected from light, and the absorbance values were measured at 570 and 630 nm using Cytation 5 (BioTek Instruments Inc., Winooski, VT, USA).

### 4.4. Cellular and Nuclear Morphology Assessment

In order to verify the potential impact of the hormonal treatment on MDA-MB-231 cells’ morphology and confluence, a microscopic evaluation was performed. The cells were observed under bright field illumination and photographed at the end of the 24 h treatment period using an Olympus IX73 inverted microscope (Olympus, Tokyo, Japan). The analysis of the photos was performed using the cellSens Dimensions v.1.8. Software (Olympus, Tokyo, Japan).

Treatment-induced changes at nuclear level are frequently associated with cytotoxicity. In this line, the Hoechst 33342 staining assay was performed to analyze the effects of EE, LNG, and EE-LNG at MDA-MB-231 cells’ nuclear level after 24 h of treatment. The experimental protocol was applied according to manufacturer’s recommendations. Briefly, the cells were cultured in 12-well plates at a density of 1 × 10^5^ cells/1.5 mL medium/well and treated with two selected concentrations (0.05 and 10 µM) of EE, LNG, and EE-LNG in DMSO for 24 h. After the stimulation period, the old media that contained the test compounds was removed and 0.5 mL of the staining solution (1:2000 in PBS) was added to each well. The cells were incubated for 10 min at room temperature, protected from light. The final steps consisted of the removal of the staining solution, washing with PBS, and taking pictures using Cytation 1 (BioTek Instruments Inc., Winooski, VT, USA). The analysis of the images was performed by the means of Gen5™ Microplate Data Collection and Analysis Software (BioTek Instruments Inc., Winooski, VT, USA). Staurosporine 5 µM (incubation for 3 h at 37 °C) was used as positive control for apoptosis, and Triton X-100 0.5% (incubation for 30 min at 37 °C) for necrosis.

### 4.5. Wound Healing Assay and Quantitative Polymerase Chain Reaction (RT-qPCR)

The influence of oral contraceptives on the migratory character of the MDA-MB-231 cells was evaluated by the means of a scratch (wound healing) assay. In brief, a number of 10^5^ cells/well were cultured in 24-well Corning plates, and when the suitable confluence (~90–95%) was reached, an automatic scratch was performed in the middle of the wells using the AutoScratch™ Wound Making Tool provided by BioTek^®^ Instruments Inc., Winooski, VT, USA. The protocol was performed as recommended by the manufacturer. After the proper wash of the wells, they were treated with the test compounds (EE, LNG, and EE-LNG solutions) at concentrations of 0.05 and 1 µM. Representative images (4x magnification) were recorded at the beginning of the test (0 h) and at its end (24 h) using Cytation 1 and were analyzed using Gen5 ™ Microplate Data Collection and Analysis Software (BioTek^®^ Instruments Inc., Winooski, VT, USA). In order to quantify the effect of the contraceptives in terms of cell migration, the difference between the initial and after 24 h wound widths was determined. The migration rate (%) was calculated according to a formula described previously by Felice et al. [67]:

(1) $$\mathrm{Scratch}\;\mathrm{Closure}\;\mathrm{Rate}\;\left(\%\right)=\frac{{\mathrm{At}}_{0}-\mathrm{At}}{{\mathrm{At}}_{0}}\times 100,\;\mathrm{where}:$$

${\mathrm{At}}_{0}$ represents the scratch area at 0 h;

At represents the correspondent scratch area at 24 h.

To evaluate the impact of OCs on gene expression, a reverse-transcription polymerase chain reaction (RT-PCR) was performed. Briefly, the MDA-MB-231 cells were cultured in 6-well plates ($10^{6}$ cells/well) and treated with EE, LNG, and EE-LNG solutions in DMSO for 48 h. Two concentrations were tested—0.05 and 10 µM. At the end of the stimulation period, the cells were mechanically detached from the wells and preserved at −20 °C as pellets for further use. The RNA was isolated from the MDA-MB-231 cells stimulated for 48 h with the test compounds (EE, LNG, and EE-LNG—0.05; 0.5 and 10 µM) using the Trizol reagent (Thermo Fisher Scientific, Inc., Waltham, MA, USA) and the Quick-RNA™ purification kit (Zymo Research). The total RNA (1 µg) was further transcribed using the Maxima^®^ First Strand cDNA Synthesis Kit (Fermentas). Quantitative real-time PCR analysis was conducted by the means of Quant Studio 5 real-time PCR system (Thermo Fisher Scientific, Inc.) in the presence of Power SYBR-Green PCR Master Mix (Thermo Fisher Scientific, Inc.). The following primer pairs (Eurogentec) were used:

18S (housekeeping gene): F: 5′-GTA-ACC-CGT-TGA-ACC-CCA-TT-3′; R: 5′-CCA-TCC-AAT-CGG-TAG-TAG-CG-3′,

Vimentin: F: 5′-CTC-TTC-CAA-ACT-TTT-CCT-CCC-3′; R: 5′-AGT-TTC-GTT-GAT-AAC-CTG-TCC-3′.

### 4.6. Statistical Analysis

The experimental data were expressed as means ± SD, and the difference between the means was compared by one-way ANOVA, followed by Dunnett’s multiple comparison post hoc test (GraphPad Prism v. 6.0 Software, San Diego, CA, SUA). The difference between groups was considered statistically significant if *p* < 0.05 and are marked with * (* *p* <0.05, ** *p* < 0.01, *** *p* < 0.00,1 and **** *p* < 0.0001).

## 5. Conclusions

The present study was meant to investigate the potential influence of 17β-Ethinylestradiol, Levonorgestrel, and their association on the behavior of the highly aggressive MDA-MB-231 hormone-independent breast cancer cells. The results were highly dependent on the exposure time, test compound, and concentration. At 24 h, cytotoxic events such as reduced proliferation, altered cell morphology, inhibited cell migration and apoptosis were noticed. The most active compound in terms of cytotoxicity was EE which exhibited a dose-dependent action. LNG showed a stimulatory trend as regards the cell survival, while EE-LNG behaved similarly to EE. At 48 h, all samples increased the cell viability and number. EE and LNG upregulated the expression of vimentin, suggesting a stimulation in cell migration, while in combination, they downregulated the gene expression which can be associated with an anti-migratory effect. Further studies are required to clarify the molecular mechanisms involved in the behavioral changes induced in TNBC cells as response to 17β-Ethinylestradiol and Levonorgestrel. However, these results open a pathway regarding the impact of OCs on triple-negative breast cancer.

## Data Availability

Not applicable.

## Sample Availability

Samples of the compounds are not available from the authors.
